# PI3Kδ inhibition elicits anti-leukemic effects through Bim-dependent apoptosis

**DOI:** 10.1038/leu.2016.333

**Published:** 2016-12-20

**Authors:** M J Carter, K L Cox, S J Blakemore, A H Turaj, R J Oldham, L N Dahal, S Tannheimer, F Forconi, G Packham, M S Cragg

**Affiliations:** 1 Antibody and Vaccine Group, Cancer Sciences Unit, Faculty of Medicine, University of Southampton, Southampton General Hospital, Southampton, UK; 2 Cancer Research UK Centre, Cancer Sciences Unit, Faculty of Medicine, University of Southampton, Southampton, UK; 3 Gilead Sciences Inc., Foster City, CA, USA

## Abstract

PI3Kδ plays pivotal roles in the maintenance, proliferation and survival of malignant B-lymphocytes. Although not curative, PI3Kδ inhibitors (PI3Kδi) demonstrate impressive clinical efficacy and, alongside other signaling inhibitors, are revolutionizing the treatment of hematological malignancies. However, only limited *in vivo* data are available regarding their mechanism of action. With the rising number of novel treatments, the challenge is to identify combinations that deliver curative regimes. A deeper understanding of the molecular mechanism is required to guide these selections. Currently, immunomodulation, inhibition of B-cell receptor signaling, chemokine/cytokine signaling and apoptosis represent potential therapeutic mechanisms for PI3Kδi. Here we characterize the molecular mechanisms responsible for PI3Kδi-induced apoptosis in an *in vivo* model of chronic lymphocytic leukemia (CLL). *In vitro*, PI3Kδi-induced substantive apoptosis and disrupted microenvironment-derived signaling in murine (Eμ-Tcl1) and human (CLL) leukemia cells. Furthermore, PI3Kδi imparted significant therapeutic responses in Eμ-Tcl1-bearing animals and enhanced anti-CD20 monoclonal antibody therapy. Responses correlated with upregulation of the pro-apoptotic BH3-only protein Bim. Accordingly, Bim^−/−^ Eμ-Tcl1 Tg leukemias demonstrated resistance to PI3Kδi-induced apoptosis were refractory to PI3Kδi *in vivo* and failed to display combination efficacy with anti-CD20 monoclonal antibody therapy. Therefore, Bim-dependent apoptosis represents a key *in vivo* therapeutic mechanism for PI3Kδi, both alone and in combination therapy regimes.

## Introduction

Secondary lymphoid organs (SLOs) provide a key survival niche for neoplastic B-cells. Here cells receive a milieu of pro-survival signals, including those emanating from the B-cell receptor (BCR), chemokine/cytokine receptors, integrins and specific cell–cell interactions. Collectively, these pathways contribute toward malignant cell proliferation, survival and therapeutic resistance.^1^

Since phosphatidylinositol-3 kinase (PI3K) signaling is vital in many of these processes, its inhibition represents an attractive therapeutic strategy. Class I PI3Ks represent a prime target in hematological malignancies due to their roles in linking cell surface receptors to downstream kinase activation in lymphocytes (for example Akt and Btk).^2, 3^ PI3Ks are heterodimeric, comprising a p110 catalytic subunit and a p85 regulatory subunit. Mammalian systems exhibit multiple isoforms of class I PI3K catalytic subunits (namely, p110α, p110β, p110δ and p110γ), which display tissue-specific expression patterns and non-redundant roles in development.^4^ Both p110α and p110β are expressed ubiquitously,^5, 6^ whereas p110δ and p110γ are largely leukocyte restricted.^4^

Accordingly, mice deficient in p110δ (referred to as PI3Kδ henceforth) activity exhibit profound disruption of lymphocyte homeostasis and humoral immunity^7^ via effects centered upon antigen receptor signaling,^7, 8^ cytokine production^8, 9^ and T_reg_ function.^10^ Consequently, δ isoform selective PI3K inhibitors (PI3Kδi) have provided encouraging therapeutic responses in clinical trials,^11^ particularly in combination with anti-CD20 monoclonal antibodies (mAb),^12^ culminating in the approval of Zydelig (idelalisib) for the treatment of relapsed refractory CLL in combination with rituximab. Although the therapeutic potential of these agents is unquestionable, the exact *in vivo* therapeutic mechanism remains ambiguous. With the ever-increasing number of novel therapeutic agents, the challenge is to identify the most efficacious, potentially curative, drug combinations. A clear mechanistic understanding of how these agents work will help provide a rational framework for improved efficacy and the circumvention of resistance mechanisms, which have emerged for other small molecule inhibitors.^13^

Potential PI3Kδi *in vivo* effector mechanisms can be stratified into those influencing the malignant cell directly (intrinsic) and those mediating effects on the host immune system (immunomodulatory effects). The latter occurs through T_reg_ suppression, resulting in enhanced anti-tumor immunity in solid tumor models.^10^ In contrast to solid tumors, PI3Kδ is often expressed within malignant lymphocytes themselves; therefore, additional malignant cell intrinsic mechanisms are likely to exist in hematological cancers. These include inhibition/alteration of tissue homing,^14^ microenvironment-derived support^15, 16^ and BCR-mediated survival signals.^14^

It is likely that these effects are integrated *in vivo* and collectively modulate malignant cell survival through regulation of intrinsic apoptosis.^17, 18^ Intrinsic apoptosis is regulated by members of the Bcl-2 family. Under normal conditions, the pro-apoptotic activities of activated Bax/Bak are repressed via association with pro-survival Bcl-2 family members (Bcl-2, Bcl-X_L_, Bcl-w, Mcl-1 and Bfl-1/A1).^19^ Following apoptotic stimuli, pro-survival molecules are inhibited by association with pro-apoptotic BH3-only proteins (Bad, Bid, Bik, Bim, Bmf, Hrk, Noxa and Puma) and Bax/Bak subjected to further activation by a subset of these proteins.^20, 21^ Subsequently, cell death ensues following saturation of pro-survival molecules and de-repression of activated Bax/Bak.^21^

Bim is a major regulator of immune homeostasis, since Bim^−/−^ animals exhibit expanded lymphocyte populations and increased autoreactivity.^22, 23^ In B-cells, this homeostatic control manifests through BCR-mediated upregulation of Bim expression during immature B-cell negative selection,^22^ although additional BH3-only proteins also contribute.^24^ Furthermore, BCR signals maintain mature B-cell populations via a PI3K-dependent mechanism, which involves suppression of Bim.^25^ Similarly, soluble factors CXCL12, BAFF and APRIL elicit their pro-survival effects either through suppression of Bim^26, 27, 28^ or increased expression of pro-survival Bcl-2 family members.^29^

On the basis of the key role of PI3Kδ in these processes, we hypothesized that PI3Kδi disrupt multiple pro-survival inputs culminating in Bim-mediated intrinsic apoptosis and *in vivo* clearance of malignant cells. Although prior studies have been performed assessing PI3Kδi-mediated immunomodulation, only limited data are available assessing the impact of PI3Kδ inhibition within a malignant target *in vivo*. Using primary human and mouse (Eμ-Tcl1 Tg) CLL cells alongside a highly selective PI3Kδi, GS-9820,^30^ we demonstrate that intrinsic apoptosis driven by Bim is the central *in vivo* therapeutic mechanism for PI3Kδi. This knowledge allowed the rational design of a complementary drug combination strategy incorporating inhibitors of PI3Kδ and Bcl-2 (Venetoclax). This approach proved highly efficacious *in vivo*, effectively halting leukemia progression in treated animals.

## Materials and methods

### Patients and cells

Diagnosis of CLL was according to the IWCLL-NCI 2008 criteria.^31^ Samples were studied following ethical committee approval (REC reference: 228/02/t) under the Declaration of Helsinki. Malignant cell isolation, determination of purity, and *in vitro* culturing were described previously.^32^ Eμ-Tcl1 Tg leukemias were isolated from splenocytes by density gradient centrifugation and maintained in RPMI-1640 supplemented with 10% fetal calf serum, 1 mM pyruvate, 2 mM glutamine, 45 units/ml penicillin, 45 μg/ml streptomycin (Thermo Fisher, Loughborough, UK), 50 μM 2-mercaptoethanol and 200 μM  L-asparagine (Sigma, Gillingham, UK). Samples exhibiting >85% CD5^+^B220^+^ cells were used directly. Samples with <85% CD5^+^B220^+^ cells were first purified using a Mouse Pan B-Cell Isolation kit (Miltenyi Biotec, Bisley, UK).

### Animals

Animals were maintained in local facilities and experiments approved by local ethical committees under Home Office license PPL30/2964. Eμ-Tcl1 Tg mice^33^ were a gift from Dr Egle (Salzburg Cancer Research Institute, Salzburg, Austria) following permission from Dr Pekarsky and Professor Croce (Ohio State University, Ohio, USA). Eμ-Tcl1 Tg animals were crossed with Bim^−/−^ animals (Jackson Laboratory, Bar Harbor, ME, USA) or Vav-Bcl-2 Tg animals (obtained from Professor Hacker following permission from Professor Adams). Animals were monitored for disease presentation by monthly blood sampling and CD5 × B220/CD19 flow cytometry. Animals were killed once 2 out of 3 criteria were met: (1) CD5^+^ B-cells >80% of circulating lymphocytes, (2) spleen (determined by palpation) >30 mm and (3) total white blood cell (WBC) counts >5 × 10^7^/ml. In transplant experiments sex-matched severe combined immunodeficiency syndrome (SCID) mice (Charles River, Saint-Germain-Nuelles, France) were inoculated with 1 × 10^7^ Eμ-Tcl1 Tg splenocytes and monitored for disease presentation. Animals were killed when 2 out of 3 criteria were met: (1) spleen >30 mm, (2) WBC counts >5 × 10^7^/ml and (3) illness requiring euthanasia.

### Antibodies and inhibitors

Anti-mouse CD20 (Clone: 18B12) mouse IgG2a was produced in-house. 18B12 was administered *in vivo* by intraperitoneal injection of 250 μg in phosphate-buffered saline. Anti-CD20-mediated opsonization of human targets was performed using Rituximab, which was gifted by Southampton General Hospital oncology pharmacy. GS-9820 was provided by Gilead Sciences Inc., (Foster City, CA, USA) and administered *in vivo* at 10 mg/kg formulated in 0.5% methylcellulose 0.1% Tween-80 *per os, bis in die* (BID). ABT-199 was administered *in vivo per os* by formulation in 60% Phosal-50 PG (Lipoid, Ludwigshafen, Germany), 30% PEG-400, 10% ethanol at 50 mg/kg for 7 days followed by 100 mg/kg for a further 14 days. Further inhibitors and antibodies utilized are detailed in the Supplementary methods.

### Cellular assays

Annexin V/propidium iodide (PI) assays were performed as previously described.^24^ For further details and other methods see Supplementary methods.

### Statistics

Data were expressed as mean±s.e.m. Statistical significance was assessed by Student's *t*-test or two-way ANOVA. Survival was assessed via Mantel–Cox statistical analysis.

## Results

### PI3Kδ inhibition disrupts BCR-induced signaling and adhesion in Eμ-Tcl1 Tg leukemias

To investigate the *in vivo* therapeutic mechanism of PI3Kδi, we utilized the Eμ-Tcl1 Tg model, a gold standard for aggressive CLL.^33, 34^ Consistent with previous reports^35, 36^ our cohort presented with a CD5^+^ B-cell leukemia with phenotypic hallmarks of CLL, extensive splenomegaly and a median survival of 294 days (Supplementary Figures 1–2). To inhibit PI3Kδ, we utilized the PI3Kδi GS-9820 (Supplementary Figure S3), recently the subject of clinical trials,^37^ as it has excellent isoform specificity^30^ and better pharmacokinetic properties than idelalisib in the mouse (Gilead unpublished observations and Supplementary Figure 3).

**Figure 1:**
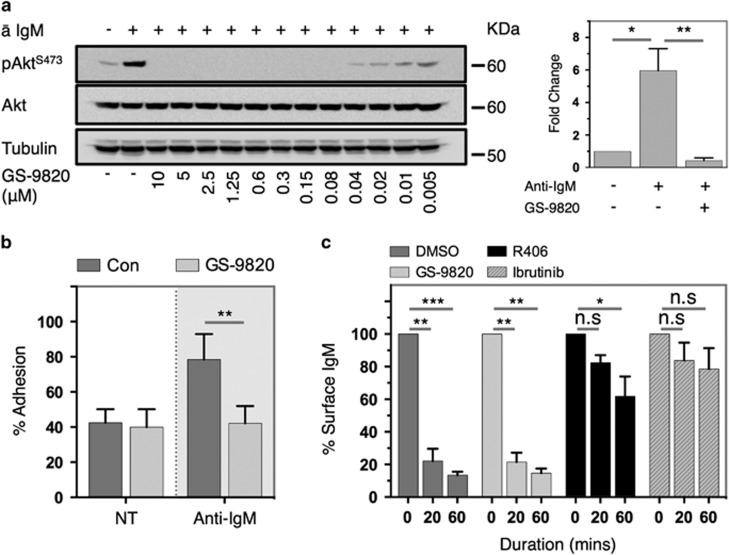
Inhibition of PI3Kδ disrupts BCR-induced signaling and adhesion in Eμ-Tcl1 Tg leukemias. (**a**) Eμ-Tcl1 Tg leukemia cells were pre-incubated with the indicated concentration of GS-9820 for 1 h followed by stimulation with 20 μg/ml anti-IgM for 3 min. Akt phosphorylation status was subsequently assessed by western blot. Left panel: representative example; right panel: densitometry values from six independent tumors utilizing 0.6 μM GS-9820. Data represented as fold change in pAkt levels over baseline. (**b**) Eμ-Tcl1 Tg leukemia cells were pre-incubated with 0.6 μM GS-9820 for 1 h before stimulation with anti-IgM. Adhesion to fibronectin-coated plates was then assessed 3 h later. Data represents an average of seven independent experiments utilizing different tumors. Adherent cell numbers are expressed as a proportion of a poly-L-lysine-coated maximum adhesion control. NT denotes non-treated. (**c**) Eμ-Tcl1 Tg leukemia cells were pre-incubated with 0.6 μM GS-9820, 1 μM R406 or 1 μM ibrutinib for 1 h before stimulation with anti-IgM. Cells were opsonized with anti-IgM on ice and levels of antibody retained at the cell surface monitored following warming to 37 °C by indirect flow cytometry. Median fluorescence intensity values were normalized to cells on ice before warming. Data represents an average of three independent experiments using different tumors. Error bars represent s.e.m. Data analyzed using an unpaired Student's *t*-test. *P<0.05, **P<0.005, ***P<0.0005, n.s.=non-statistically significant.

BCR stimulation increased phosphorylation of the PI3K-target Akt at both S473 and T308, which was inhibited by prior application of GS-9820 in a dose-dependent manner (Figure 1a and Supplementary Figure 4a). Concurrent inhibition of ERK, but not Syk, phosphorylation was also evident (Supplementary Figure 4b). GS-9820 also significantly inhibited BCR-induced adhesion to fibronectin (Figure 1b), yet in contrast to inhibitors of Btk (ibrutinib) and Syk (R406) had no impact on BCR internalization (Figure 1c). These data indicate that GS-9820 effectively inhibits BCR-induced kinase signaling and impairs some functional consequences of BCR stimulation.

### PI3Kδ inhibition impairs chemotaxis and pro-survival responses to microenvironment-derived factors in Eμ-Tcl1 Tg leukemias

**Figure 2:**
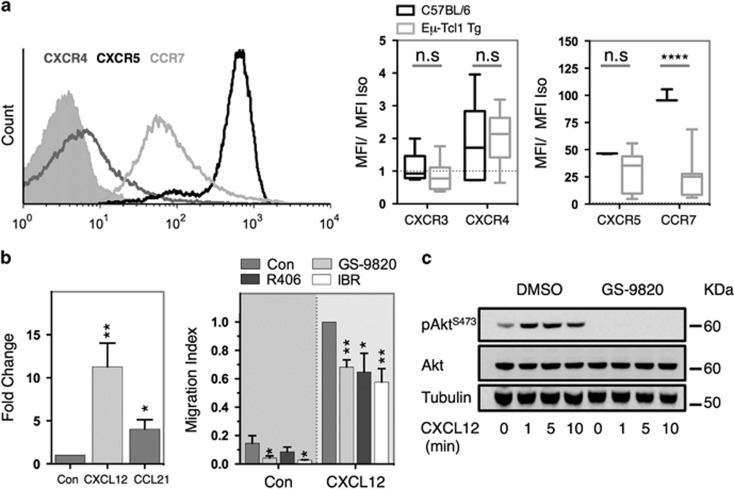
GS-9820 inhibits chemotaxis and chemokine-dependent signaling. (**a**) Surface chemokine receptor expression was assessed in both Eμ-Tcl1 Tg leukemias and normal C57BL/6 B-cells by flow cytometry. Representative data depicted left. Data, expressed as median fluorescence intensity/ median fluorescence intensity isotype control (filled histogram), represent averages of 10 different tumor and 5 C57BL/6 B-cell populations. (**b**) CXCL12- (200 ng/ml) or CCL21- (1 μg/ml) mediated chemotaxis of Eμ-Tcl1 Tg leukemias was assessed in transwell assays (left) and the impact of kinase inhibition (0.6 μM GS-9820, 1 μM R406, 1 μM ibrutinib (IBR)) assessed in comparison to a DMSO control (Con) (right). Data were normalized to medium alone (left) or CXCL12 chemotaxis in DMSO-treated cells (right) and represent averages of seven different tumors. (**c**) The impact of GS-9820 (0.6 μM) on CXCL12-mediated (200 ng/ml) Akt phosphorylation was assessed by western blot. Data representative of independent experiments using two different tumors. Data analyzed using unpaired (**a**) or paired (**b**) Student's *t*-test. Error bars represent s.e.m. *P<0.05, **P<0.005, ****P<0.00005, n.s.=non-significantly different.

To assess the impact of GS-9820 on cellular migration and tissue homing responses, Eμ-Tcl1 Tg leukemias were examined for surface expression of chemokine receptors. Eμ-Tcl1 Tg leukemias exhibited heterogeneous surface staining for CXCR4, CXCR5 and CCR7, but were negative for CXCR3 (Figure 2a). Although CXCR4 and CXCR5 expression was similar to that of normal B-cells, CCR7 was significantly reduced. Accordingly, both CXCL12 and CCL21 induced transmigration of Eμ-Tcl1 Tg leukemias in transwell assays (Figure 2b). GS-9820, R406 and ibrutinib inhibited, but did not ablate, CXCL12-mediated chemotaxis, as previously identified (Figure 2b).^14, 38, 39^ Consistent with this, GS-9820 inhibited CXCL12-induced phosphorylation of Akt^S473^ (Figure 2c), yet in contrast to BCR signaling was unable to concomitantly inhibit ERK phosphorylation (Supplementary Figure 4c). These data suggest that CXCL12-mediated chemotaxis and downstream kinase activation is only partially PI3Kδ dependent in Eμ-Tcl1 Tg leukemias.

**Figure 3:**
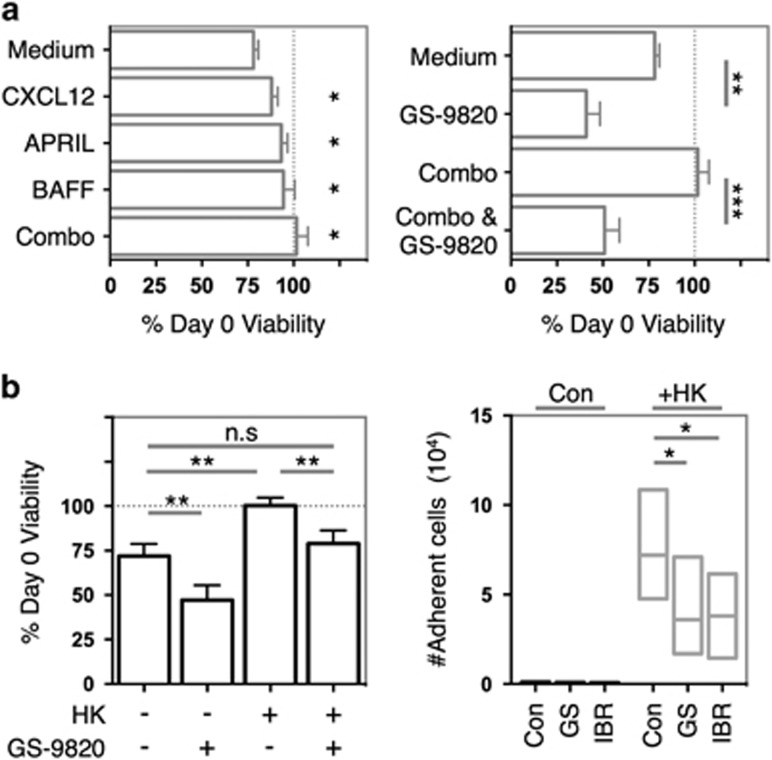
GS-9820 inhibits soluble factor- and HK cell-mediated microenvironmental support. (**a**) *Ex vivo* Eμ-Tcl1 Tg leukemias were cultured alongside CXCL12 (500 ng/ml), BAFF (200 ng/ml), APRIL (500 ng/ml) or combinations of all three (Combo) in the absence (left) or presence (right) of GS-9820 (0.6 μM) and viability assessed by Annexin V/PI flow cytometry after 5 days. Data were normalized to day 0 viability and represent averages of eight different tumors. (**b**) Left: Eμ-Tcl1 Tg leukemia: HK co-cultures were established before GS-9820 (0.6 μM) application. Viability was assessed 3 days later by Annexin V/PI flow cytometry. Right: Eμ-Tcl1 Tg leukemias were pre-incubated with GS-9820 (0.6 μM) or ibrutinib (IBR) (1 μM) before HK co-culture and cell–cell contact formation assessed by flow cytometry. Data represent averages of seven different tumors. Data analyzed using paired Student's *t*-test. Error bars represent s.e.m. *P<0.05, **P<0.005, ***P<0.0005, n.s.=non-significantly different.

Since many stroma-derived soluble factors and cellular interactions provide support signals to malignant B-cells, we examined the ability of GS-9820 to impair these processes. CXCL12, BAFF, APRIL or a combination of all three significantly improved the viability of Eμ-Tcl1 Tg leukemias 5 days post *ex vivo* culture in comparison to medium alone and were effectively antagonized by GS-9820 (Figure 3a). GS-9820 application also inhibited support arising from the follicular dendritic cell line HK. For both CLL and Eμ-Tcl1 cells co-culture with HK cells enhanced viability (Figure 3b, left and Supplementary Figure 4d). This survival advantage was disrupted by addition of GS-9820 after the formation of cell–cell contacts in these co-culture settings (Figure 3b, left and Supplementary Figure 4d). In addition, both GS-9820 and ibrutinib inhibited cell–cell contact formation and adhesion when added before co-culture (Figure 3b, right). These data imply that both cytokine/chemokine and cell–cell contact-mediated support mechanisms are PI3Kδ dependent in this system.

### PI3Kδ inhibition reduces cellular viability coincident with upregulation of Bim

**Figure 4:**
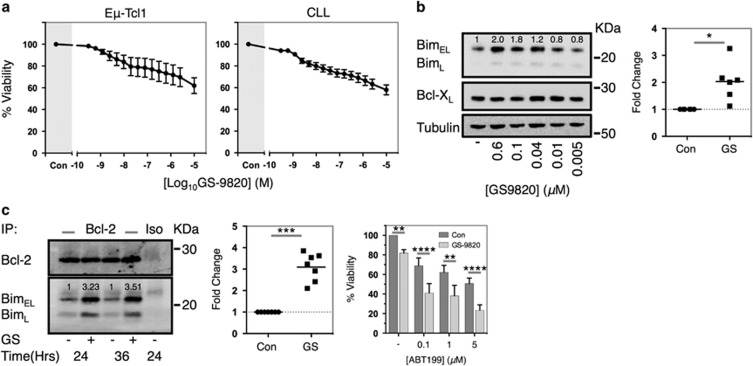
GS-9820 reduces cellular viability coincident with upregulation of Bim in Eμ-Tcl1 Tg leukemias. (**a**) Eμ-Tcl1 Tg leukemias (left) or primary human CLL (right) were cultured in the presence of GS-9820 or a DMSO control (Con) for 48 h and assessed for viability by Annexin V/PI flow cytometry. Data represent an average of 5 Eμ-Tcl1 Tg leukemias and 14 CLL samples. (**b**) Eμ-Tcl1 Tg leukemia cells were cultured alongside GS-9820 or a DMSO control (Con) for 36 h followed by western blot analysis. Left: example blot. Right: densitometry values of total Bim expression in GS-9820 (0.6 μM) (GS) treated cells normalized to vehicle control (Con) expressed as fold change from six different tumors. (**c**) Eμ-Tcl1 Tg leukemias were cultured for 24 or 36 h alongside GS-9820 (0.6 μM) (GS) or vehicle control and lysed. Lysates were then subjected to immunoprecipitation with an anti-Bcl-2 mAb or an isotype control and the extent of Bim co-immunoprecipitation assessed by western blot. Left: example blot. Center: densitometry data representing the relative fold change in Bim co-immunoprecipitation after 24 h. Data are normalized to vehicle-treated cells and expressed as fold change from control treatment. (**c**, right) Eμ-Tcl1 Tg leukemias were cultured in the presence of GS-9820 (0.6 μM) or a DMSO control for 24 h followed by application of ABT-199 for a further 24 h in the presence of 0.6 μM GS-9820 or a DMSO control. Viability was assessed by Annexin V/PI flow cytometry. Data represent an average of six different tumors, bars represent s.e.m. Statistical analysis was performed via paired Student's *t*-test analysis. *P<0.05, **P<0.005, ****P<0.00005.

Previously, PI3Kδi have been reported to reduce the *in vitro* viability of malignant B-cells, presumably through BCR inhibition.^18^ GS-9820 reduced cellular viability in both Eμ-Tcl1 Tg leukemias and CLL samples (Figure 4a); albeit with significant heterogeneity in individual Eμ-Tcl1 Tg tumors. This reduction in viability was coincident with upregulation of Bim expression and Bim–Bcl-2 complex formation, indicative of enhanced Bim function (Figures 4b and c). Similar effects were also observed in some, but not all, CLL samples and diffuse large B-cell lymphoma cell lines (Supplementary Figures 5 and 7).

High pro-survival occupancy with BH3-only proteins sensitizes cells to subsequent apoptotic stimuli due to a reduced capacity to neutralize activated Bax/Bak, known as mitochondrial priming.^40^ Because of enhanced mitochondrial priming through Bim upregulation, we hypothesized that GS-9820-treated cells would exhibit enhanced sensitivity toward the Bcl-2 inhibitor ABT-199. Indeed, administration of GS-9820 24 h before ABT-199 (to allow Bim upregulation) significantly enhanced cytotoxicity in comparison to ABT-199 treatment alone in Eμ-Tcl1 Tg leukemias, CLL and GS-9820-sensitive diffuse large B-cell lymphoma cell lines (Figure 4c and Supplementary Figures 5 and 7).

In comparison to normal B-cells, Eμ-Tcl1 Tg leukemias exhibited extensive Bcl-2 and Bcl-X_L_ expression, with little or no expression of Mcl-1, Bfl-1/A1 or Bcl-w (Supplementary Figure 8). Comparable levels of Bad and Bid were seen in Eμ-Tcl1 Tg leukemias and normal B-cells. Puma was absent in both, only upregulated following treatment with etoposide and concurrent with p53 induction, demonstrating the integrity of the TP53 pathway in Eμ-Tcl1 Tg leukemias (Supplementary Figure 8c).^41^ However, significant heterogeneity in basal Bim expression was evident in Eμ-Tcl1 Tg leukemias in comparison to normal B-cells, with the level predictive of overall sensitivity to GS-9820-induced death (Supplementary Figures 8a and b). These data indicate a direct link between PI3Kδ (and its inhibition) and the regulation of Bim.

### Genetic loss of Bim does not alter Tcl1-induced leukemiagenesis

**Figure 5:**
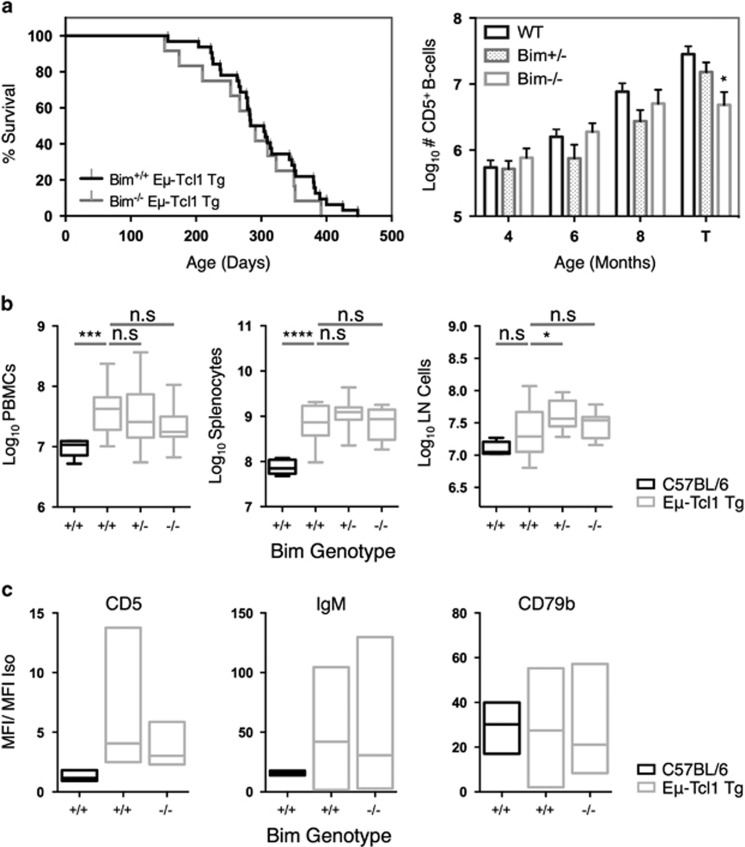
Genetic loss of Bim does not accelerate nor exacerbate Tcl1-induced leukemiagenesis. (**a**) Eμ-Tcl1 Tg animals were crossed onto a Bim^−/−^ background and monitored for survival (left) and the number of circulating leukemia cells (right) by flow cytometry. T, terminal disease. Survival data represent sample groups of *n*=32 Bim^+/+^ Eμ-Tcl1 Tg and *n*=12 Bim^−/−^ Eμ-Tcl1 Tg animals. (**b**) Eμ-Tcl1 Tg animals were culled and organs collected once terminal disease status was reached. Cell counts were performed on the blood (left), spleen (center) and lymph nodes (right) of Bim^+/+^, Bim^+/–^, or Bim^−/−^ Eμ-Tcl1 Tg animals (gray bars) and compared with aged C57BL/6 animals (black bars). Cell count data represent median counts obtained from 8 C57BL/6, 15 Bim^+/+^ Eμ-Tcl1 Tg, 17 Bim^+/−^ Eμ-Tcl1 Tg and 9 Bim^−/−^ Eμ-Tcl1 Tg animals. (**c**) The surface phenotype of splenocytes derived from terminal Eμ-Tcl1 Tg animals were assessed by flow cytometry. Data were obtained from CD5^+^ B220^+^ gated cells and expressed as a ratio with the median fluorescence intensity of an appropriate isotype control. Data represent an average of values obtained from 16 different Eμ-Tcl1 Tg leukemias, 7 Bim^−/−^ Eμ-Tcl1 Tg leukemia and 5 C57BL/6 B-cell populations. Survival analysis was performed using a log-rank test and cell count data interpreted using an unpaired Student's *t*-test. *P<0.05, ***P<0.0005, ****P<0.00005, n.s.=non-statistically different.

Previously, Bim has been identified as a key tumor suppressor in both murine and human cancers, with reduced expression associated with therapeutic resistance.^42, 43, 44^ To further assess the role of Bim in Tcl1-mediated leukemiagenesis and PI3Kδi therapy, Eμ-Tcl1 Tg animals were crossed with Bim^−/−^ animals. Bim^−/−^ Eμ-Tcl1 Tg animals exhibited an equivalent median survival to Bim^+/+^ Eμ-Tcl1 Tg albeit with a reduced leukemic burden at sacrifice (Figure 5a). Nonetheless, peripheral blood mononuclear cell, splenocyte and lymph node cell numbers were largely comparable (Figure 5b). Western blot revealed that Bim^−/−^ Eμ-Tcl1 Tg leukemias exhibited comparable levels of Bid, Puma, Bcl-2 and Bcl-X_L_ expression to their Bim^+/+^ counterparts (Supplementary Figure 9a) indicating that compensatory changes in expression of activator BH3-only proteins, or pro-survival molecules, had not occurred and that the tumors did not display stabilizing p53 mutations. Furthermore, leukemias arising in Bim^−/−^ Eμ-Tcl1 Tg animals exhibited an equivalent immunophenotype, to their Bim^+/+^ Eμ-Tcl1 Tg counterparts (Figure 5c and Supplementary Figure 9b). Overall, these data indicate that Bim does not function as a tumor suppressor in this model.

### Bim^−/−^ Eμ-Tcl1 Tg leukemias respond normally to PI3Kδ-dependent stimuli but are resistant toward PI3Kδi-induced cytotoxicity

**Figure 6:**
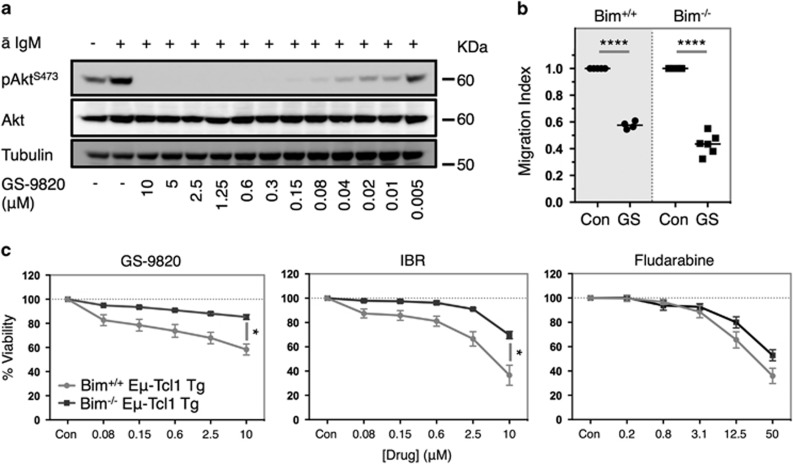
Bim^−/−^ Eμ-Tcl1 Tg leukemias remain sensitive to GS-9820-mediated inhibition of BCR signaling and CXCL12-mediated chemotaxis but are refractive to cell death induced by GS-9820 and ibrutinib. (**a**) Bim^−/−^ Eμ-Tcl1 Tg leukemia cells were pre-incubated with GS-9820 or a DMSO control for 1 h followed by stimulation with anti-mouse IgM (20 μg/ml) for 3 min. Akt phosphorylation status was subsequently assessed by western blot. Data are representative example of two independent experiments utilizing different Bim^−/−^ tumors. (**b**) Eμ-Tcl1 Tg leukemias from both Bim^+/+^ and Bim^−/−^ background were pre-incubated with GS-9820 (0.6 μM) (GS) for 1 h and migration toward CXCL12 (200 ng/ml) assessed using a transwell migration assay. Data were normalized to CXCL12-mediated migration in control-treated cells and expressed as a ratio. Data represent an average of values obtained from four different Bim^+/+^ and six different Bim^−/−^ tumors. (**c**) Bim^+/+^ and Bim^−/−^ Eμ-Tcl1 Tg leukemias were subjected to the indicated concentration of GS-9820, ibrutinib (IBR), fludarabine or a DMSO control (Con) for 48 h and assessed by Annexin V/PI flow cytometry for viability. Data represent the average of values obtained from 12 Bim^+/+^ and 5 Bim^−/−^ Eμ-Tcl1 Tg leukemias. Cell migration data were analyzed by paired Student's *t*-test, whereas viability assays were assessed by two-way ANOVA. *P<0.05, ****P<0.00005.

Before assessing the impact of loss of Bim on GS-9820-induced cytotoxicity, we first assessed the integrity of PI3K-dependent pathways in Bim^−/−^ Eμ-Tcl1 Tg leukemias and their sensitivity to GS-9820. Like their wild-type counterparts, Bim^−/−^ Eμ-Tcl1 Tg leukemias exhibited enhanced Akt^S473^ phosphorylation following BCR stimulation, which was ablated by GS-9820 in a dose-dependent manner (Figure 6a). Furthermore, CXCL12-mediated chemotaxis remained intact and sensitive to GS-9820 inhibition (Figure 6b). In contrast, genetic loss of Bim imparted significant resistance toward GS-9820- and ibrutinib-induced cytotoxicity in Eμ-Tcl1 Tg tumors, whereas responses toward fludarabine were less affected (Figure 6c). Although significantly inhibited, residual levels of GS-9820- and ibrutinib-induced cell death were evident in Bim^−/−^ leukemias. This residual activity was absent in Eμ-Tcl1 Tg leukemias over-expressing Bcl-2 (Supplementary Figure 10) derived from vav-Bcl-2 Tg Eμ-Tcl1 Tg mice. These data demonstrate that Bim plays a major role in the cytotoxic responses induced by GS-9820 and ibrutinib with a minor contribution by other BH3-only proteins.

### PI3Kδi elicit anti-leukemic effects *in vivo* dependent on Bim

To investigate the mechanisms of PI3Kδi *in vivo*, SCID animals were inoculated with Eμ-Tcl1 Tg leukemias. These mice lack adaptive immune cells and permit the investigation of mechanisms independent of the reported effects on T_reg_.^10^

**Figure 7:**
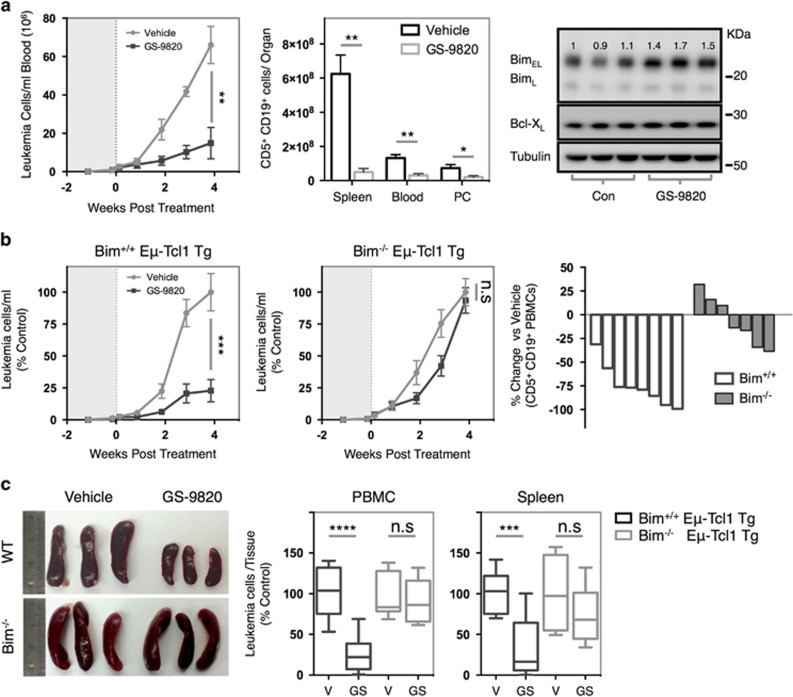
GS-9820 elicits anti-leukemic effects *in vivo* dependent upon the BH3-only protein Bim. (**a**) SCID mice were inoculated with 1x10^7^ Bim^+/+^ Eμ-Tcl1 Tg splenocytes and monitored for disease by weekly blood sampling and flow cytometry. Upon leukemia detection, animals were randomized into groups (*n*=4 per group) receiving 10 mg/kg GS-9820 or vehicle control *per os* BID and monitored for disease (left). Four weeks post treatment, vehicle-recipient animals reached terminal disease and all mice were killed, organs collected and leukemia cells within the spleen, blood and peritoneal cavity enumerated (center). Right: leukemia cells were purified from vehicle (Con) or GS-9820-treated animals and the expression of Bim assessed by western blot. Data are representative of two independent experiments performed using two different Bim^+/+^ tumors. (**b**) SCID animals were inoculated with either Bim^+/+^ or Bim^−/−^ Eμ-Tcl1 Tg tumors and leukemia-bearing animals treated with either GS-9820 or a vehicle control and monitored for disease progression as in **a** (left, Bim^+/+^; center, Bim^−/−^). Data represent averages of two independent experiments using two different tumors per genotype expressed as percentage of the maximum leukemic burden achieved in vehicle-treated mice (% control). Right: 4 weeks post treatment, the relative percentage change in CD5^+^ CD19^+^ peripheral blood mononuclear cell number was compared with GS-9820-treated mice of each genotype. (**c**) Animals from **b** were killed when vehicle-treated animals reached terminal disease and organs collected (GS=GS-9820). Gross pathology (left) and leukemic content in peripheral blood mononuclear cells (center) and spleen (right) were assessed. Data represent average values obtained from two independent experiments using two different Eμ-Tcl1 Tg leukemias per genotype with at least seven animals present per group. Data were analyzed by paired Student's *t*-test and leukemia enumeration curves were analyzed by two-way ANOVA. *P<0.05, **P<0.005, ***P<0.0005, ****P<0.00005, n.s.=non-statistically different.

Following detection of CD5^+^ B-cells in the blood, mice were treated with 10 mg/kg GS-9820 or vehicle control *per os* BID. Although PI3Kδ-mutant animals exhibit colitis,^7^ GS-9820-recipient animals did not demonstrate any adverse effects attributable to treatment (Supplementary Figure 11a). GS-9820-treated animals exhibited a 75% reduction in leukemic burden 4 weeks post administration alongside tumor reductions in the peritoneum and spleen (Figures 7a and b). Therapeutic responses to GS-9820 correlated with enhanced Bim expression (≈1.5-fold) in spleen-resident tumor cells in comparison to vehicle-recipient animals (Figure 7a, right). To dissect the role of Bim-dependent apoptosis in PI3Kδi therapy, Bim^−/−^ Eμ-Tcl1 Tg leukemias were transferred into SCID recipients and assessed for responses toward GS-9820. Bim^−/−^ Eμ-Tcl1 Tg leukemia-recipient animals appeared refractory to GS-9820, with little variation in leukemic burden, spleen deposits or splenomegaly evident (Figures 7b and c).

In addition to T_reg_ inhibition, myeloid-derived suppressor cells (MDSC) have been implicated in PI3Kδi-mediated therapeutic responses.^10^ In both Bim^+/+^ and Bim^−/−^ leukemia-recipient animals, the frequency of splenic MDSC populations (CD11b^+^ Ly6C^High^ Ly6G^Low^ monocytic-MDSC (M-MDSC) or CD11b^+^ Ly6C^Low^ Ly6G^High^ polymorphonuclear-MDSC (PMN-MDSC)) were unaffected by PI3Kδi administration (Supplementary Figure 12). Therefore, the therapeutic effects of PI3Kδi monotherapy appear largely dependent on Bim-dependent intrinsic apoptosis within the malignant lymphocyte.

### PI3Kδi enhance anti-CD20 mAb therapy in a Bim-dependent manner

Since PI3Kδi effectively combine with anti-CD20 mAb in the clinic,^12^ we determined whether this synergistic effect was dependent on Bim-induced apoptosis. Anti-CD20 mAbs induced a robust depletion of leukemic targets in Eμ-Tcl1 Tg-recipient animals that was ablated in mice lacking the FcγR γ chain^−/−^, required for activatory FcγR expression and activity (Supplementary Figure 11b). In agreement with other models^45^ these data identify activatory FcγR-mediated processes as the major anti-CD20 mAb effector mechanism in the Eμ-Tcl1 Tg model.

**Figure 8:**
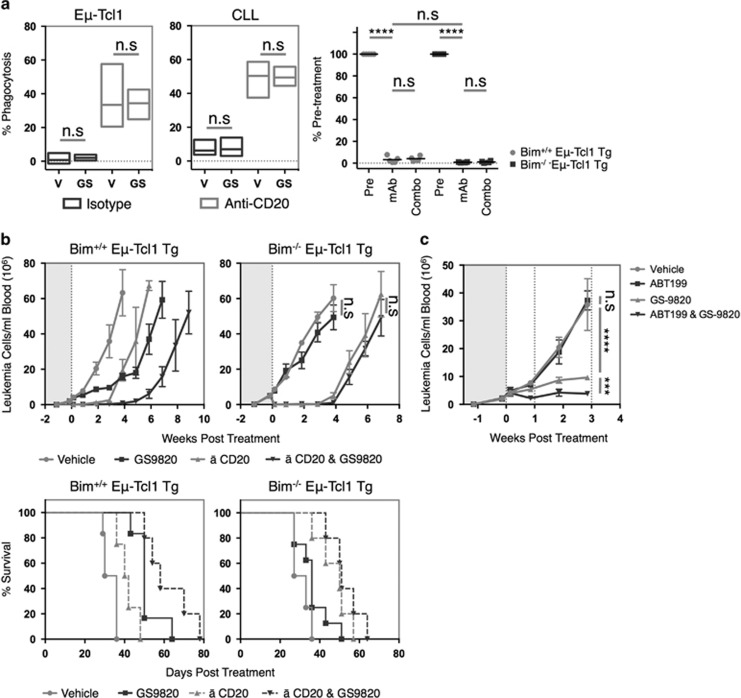
GS-9820-mediated enhancement of anti-CD20 mAb therapy is dependent on Bim. (**a**) GS-9820- (0.6 μM) or DMSO control-treated Eμ-Tcl1 Tg leukemias (*n*=3) (left) or primary human CLL (*n*=4) (center) were opsonized with anti-CD20 mAb (18B12 or rituximab) or an isotype control, co-cultured with mouse (left) or human (center) macrophages and phagocytosis assessed by flow cytometry. (Right) SCID mice were inoculated with Bim^+/+^ or Bim^−/−^ Eμ-Tcl1 Tg leukemias and randomized into treatment groups (*n*=5 per group) following presentation, receiving either anti-mouse CD20 (250 μg 18B12) and vehicle control or anti-CD20 (250 μg 18B12) and GS-9820 10 mg/kg *per os* BID. The extent of leukemia depletion was then assessed 48 h later. Data were normalized to pre-treatment levels. (**b**) SCID mice were inoculated as in **a** and randomized into treatment groups (*n*=5 per group) receiving GS-9820 (10 mg/Kg), vehicle control, anti-mouse CD20 (250 μg 18B12) and vehicle or anti-CD20 and GS-9820. Mice were maintained on therapy and monitored for disease progression and overall survival. (**c**) SCID mice were inoculated with Bim^+/+^Eμ-Tcl1 Tg leukemias, as in **b**, and randomized into treatment groups (*n*=5 per group) receiving GS-9820, ABT-199 (50-100 mg/kg), combinations of both, or a vehicle control and monitored disease progression. To assess initial sensitivity, animals were dosed with 50 mg/kg ABT-199 for 7 days and escalated to 100 mg/kg for a further 14 days. Statistical analyses were performed using a paired Student's *t*-test (**a**, **b**) or two-way ANOVA (**c**). n.s.= non-significant. ***P<0.0005, ****P<0.00005. Survival analysis was performed using a log-rank test.

Consequently, the impact of GS-9820 on antibody-dependent cellular phagocytosis was examined. Anti-CD20-mediated antibody-dependent cellular phagocytosis appeared unaffected by GS-9820 utilizing either murine or human cells (Figure 8a, left and center). Furthermore, GS-9820 did not influence maximal anti-CD20 mAb-mediated leukemia depletion 48 h post treatment of either Bim^+/+^ or Bim^−/−^ leukemia-recipient animals (Figure 8a, right). Collectively, these data indicate that augmentation of antibody-dependent cellular phagocytosis is not responsible for the combination effects of GS-9820 and anti-CD20 mAb.

Both single 250 μg dose of anti-CD20 mAb and GS-9820 monotherapy extended overall survival (*P*<0.05 and *P*<0.005, respectively) in Bim^+/+^ Eμ-Tcl1 Tg-recipient animals. Furthermore, anti-CD20 mAb and GS-9820 combination therapy enhanced the duration of anti-CD20-mediated leukemia depletion and overall survival in comparison to mAb therapy alone (*P*<0.005) (Figure 8b, left panels). In contrast, the therapeutic effect of GS-9820 was lost in both monotherapy and combination therapy treatment groups in Bim^−/−^ Eμ-Tcl1 Tg-recipient animals, whereas anti-CD20 mAb therapy appeared unaffected and enhanced overall survival in comparison to vehicle controls (*P*<0.005) (Figure 8b, right panels). These data confirm that Bim-dependent apoptosis represents the primary therapeutic mechanism of GS-9820 in both monotherapy and combination therapy regimes.

This knowledge can be employed in the design of subsequent treatment regimes. As a proof of concept, it was reasoned that combinations of GS-9820 and ABT-199 would be highly efficacious *in vivo*. Both ABT-199 monotherapy and combinations with GS-9820 appeared well tolerated, with no symptoms of toxicity associated with treatment. Although GS-9820:ABT-199 combination-treated animals maintained weight, a slight, non-significant reduction in the rate of weight gain was evident in comparison to other treatment groups (Supplementary Figure 11c). Although ABT-199 monotherapy was ineffective against Bim^+/+^ Eμ-Tcl1 Tg leukemias, combinations of GS-9820 and ABT-199 proved more effective than GS-9820 monotherapy alone (Figure 8c). This strategy reduced leukemic burden by 95% in comparison to vehicle controls, effectively sensitizing Eμ-Tcl1 Tg leukemias to ABT-199. These findings facilitate the design of novel combination therapies and potentially provide a strategy to overcome microenvironment-derived ABT-199 resistance.^46^

## Discussion

Extensive pre-clinical and clinical studies culminated in the approval of the PI3Kδi Zydelig (idelalisib) for the treatment of relapsed/refractory CLL in combination with rituximab.^10, 12, 14^ Although efficacious, this combination is not curative and primarily delays disease progression. To permit evidence-based design of potentially curative complementary drug combinations, a detailed mechanistic understanding of PI3Kδ inhibition is required. At present, *in vivo* mechanistic insights are limited to PI3Kδi-mediated immunomodulation in the treatment of solid tumors (that lack PI3Kδ expression).^10^ In this setting, PI3Kδi enhance anti-tumor immunity through T_reg_ and MDSC suppression.^10^ Since malignant lymphocytes often express PI3Kδ^4^ additional intrinsic mechanisms likely arise in lymphoid cancers. In the present study, an *in vivo* model of CLL was utilized in immunodeficient recipient animals. Within this system, animals lack adaptive immune cells (including, T_reg_) and do not exhibit PI3Kδi-dependent augmentation of MDSCs (Supplementary Figure 12), allowing the dissection of intrinsic mechanisms from immunomodulatory effects.

Previous studies suggest that tumor intrinsic mechanisms augment BCR signaling,^8, 14^ chemokine/cytokine receptor signaling^8, 14, 15^ and stromal cell support.^15, 16^ BCR signaling is inextricably linked with regulation of Bim-dependent apoptosis in both its pro-survival and pro-apoptotic signaling modes.^22, 24, 25^ In mature B-cells, BCR signals maintain B-lymphocyte populations via a PI3K-dependent mechanism linked to downregulation of Bim expression^25^ and in response to antigen, BCR signaling neutralizes Bim by MEK1-dependent phosphorylation.^47^ Conversely, in immature B-cells aberrant BCR signaling directly upregulates Bim during negative selection.^22, 24^ Like BCR signaling, many cytokines/chemokines also modulate cellular survival. In particular, BAFF, APRIL and CXCL12 are linked to concomitant downregulation of Bim and increased pro-survival expression.^27, 28, 29^ Furthermore, stromal cell interaction, particularly with follicular dendritic cells, has been identified as a major determinant of survival in CLL and the Eμ-Tcl1 Tg model, via CD44-mediated Mcl-1 upregulation and subsequent apoptotic resistance.^48, 49^ Cumulatively, these studies suggest a conserved link between BCR, cytokines/chemokines and cellular interactions in the regulation of intrinsic apoptosis and B-cell homeostasis.

Here we showed that PI3Kδi administration inhibited cellular viability (coincident with upregulation of Bim activity) and chemotaxis, alongside inhibition of BAFF-, CXCL12-, APRIL- and follicular dendritic cell-mediated survival pathways. Furthermore, PI3Kδi imparted a Bim-dependent reduction in Eμ-Tcl1 Tg leukemia cells *in vivo,* associated with increased Bim expression.

Upregulation of Bim has been observed *in vitro* with other BCR-inducible kinase inhibitors.^50, 51^ Consequently, upregulation of Bim appears a common mechanism triggered by inhibition of BCR-inducible kinases. Although PI3Kδi-mediated Bim upregulation was evident *in vitro* in the absence of exogenous antigen, antigen contamination of *in vitro* cultures from murine tissues is likely. Therefore, it remains unclear whether Bim upregulation occurs via inhibition of the BCR. In diffuse large B-cell lymphoma cell lines, where *in vitro* antigen-dependent BCR signaling is required for survival,^52^ PI3Kδi-mediated Bim upregulation was evident in some but not all cell lines. Therefore, removal of BCR-mediated Bim suppression may, at least in part, explain PI3Kδi-mediated Bim upregulation. However, since BAFF and BCR signals appear to co-operate in the maintenance of B-cell populations, via an NF-KB-centered integration node,^53^ it is likely that PI3Kδi-mediated Bim upregulation occurs as a consequence of inhibition of both BCR- and cytokine/chemokine-mediated pathways.

On the basis of the data reported herein, we propose a mechanism whereby PI3Kδ inhibition imparts therapeutic responses in hematological malignancies by both malignant cell intrinsic and immunomodulatory mechanisms. In the former, PI3Kδ inhibition abrogates BCR-mediated suppression of Bim, reducing the ability of malignant B-cells to survive in the periphery, and increasing reliance on SLO transit and survival networks. However, concurrent PI3Kδi-mediated inhibition of malignant B-cell entry into SLOs, migration to follicular dendritic cell-rich areas and inhibition of SLO-resident pro-survival signals denies this. Cumulatively, these effects result in enhanced Bim expression, enhanced mitochondrial priming and the apoptotic demise of malignant B-cells by a ‘death by neglect'-like process. Similar observations have been reported previously, whereby microenvironmental support reduced mitochondrial priming of lymph node-resident CLL cells, which was reversed on idelalisib administration.^17^

Although loss of Bim imparted significant *in vitro* and *in vivo* resistance toward PI3Kδi, therapeutic responses were not totally ablated. Because only Bcl-2 Tg Eμ-Tcl1 Tg leukemias were completely refractory to PI3Kδi-induced cytotoxicity *in vitro,* additional BH3-only proteins likely contribute. Indeed, co-operative relationships between Bim and other BH3-only proteins in hematopoiesis and responses to apoptotic stimuli are well documented, including downstream of the BCR.^24, 26^

In combination with anti-CD20 mAbs, PI3Kδi administration enhanced the duration of leukemia depletion *in vivo*, in line with clinical trial results.^12^ This effect was ablated upon genetic loss of Bim. It is likely that the enhanced duration of depletion offered by combinations of anti-CD20 mAbs and PI3Kδi reflect the Bim-mediated reduction of mAb-resistant tumor deposits within SLOs. Indeed, the anatomical distribution of target cells, and their respective access to the hepatic reticuloendothelial system, has been linked to the extent of B-cell depletion within these sites.^54^ Thus, by reducing SLO-mediated mAb resistance and cellular support mechanisms, PI3Kδi likely enhances anti-CD20 mediated depletion within SLOs and slows the rate of relapse after loss of mAb from the circulation.

Given that cytotoxicity through intrinsic apoptosis appears the primary effector mechanism of PI3Kδi *in vivo*, synergistic, or at least additive, therapeutic effects may be achieved by combining inhibitors of PI3Kδ and Bcl-2, such as ABT-199 (Venetoclax). This hypothesis is supported by our, and others, observations of enhanced apoptosis when applied in combination.^55^ Surprisingly, *in vivo* ABT-199 monotherapy proved ineffective in the treatment of Bim^+/+^ Eμ-Tcl1 Tg-recipient animals (Figure 8c). In clinical trials, ABT-199 has yielded impressive results in the treatment of relapsed/refractory CLL.^56^ This apparent ABT-199 resistance is most likely attributable to high levels of Bcl-X_L_ expression (Supplementary Figure 8a), which in CLL has been linked to a 1000-fold reduction in ABT-199 sensitivity.^46^ In contrast to ABT-199 monotherapy, PI3Kδi:ABT-199 combination therapy reduced *in vivo* leukemic burden by 95% in comparison to vehicle controls. These effects are likely attributable to enhanced Bim-mediated mitochondrial priming following PI3Kδi application allowing greater ABT-199-mediated displacement of Bim, resulting in enhanced Bax/Bak activation.

This proof-of-concept experiment demonstrates the ability of new mechanistic knowledge to provide rationale-based combination strategies for more effective treatments. Looking forward, inhibitors targeting additional aspects of microenvironmental support, such as IL-4 and CD40L, signaling could be incorporated. These T-cell-mediated microenvironment-derived support signals enhance BCR signaling and generate therapeutic resistance toward Bcl-2 inhibitors.^46, 57^ Since these pathways exhibit only partial PI3Kδ dependency^7^ use of an additional JAK:STAT inhibitor may further enhance the efficacy of PI3Kδ:Bcl-2 inhibitor combinations and provide a curative treatment regime.
