# Supplementary material for: PI3Kδ inhibition elicits anti-leukemic effects through Bim-dependent apoptosis
Source: Leukemia. 2016 Dec 20;31(6):1423–33. doi: 10.1038/leu.2016.333 (PMC5467045; doi:10.1038/leu.2016.333)
Supplement: Supplementary Methods & Figures [file leu2016333x1.docx]

**Supplementary Methods**

**Western Blot**

| Target | Species | Application | Dilution | Company | Number |
| --- | --- | --- | --- | --- | --- |
| pAkt^S473^ | Rabbit | WB | 1:500 | CST | 4060 |
| pAkt^T308^ | Rabbit | WB | 1:500 | CST | 9275 |
| Akt | Rabbit | WB | 1:500 | CST | 9272 |
| Tubulin | Rabbit | WB | 1:500 | CST | 2144 |
| Bim | Rabbit | WB | 1:500 | Enzo | ADI-AAP-330 |
| Bcl-XL | Mouse | WB | 1:500 | BD | 610747 |
| Bcl-2 | Hamster | IP/WB | 1:500/  1 μg (IP) | BD | 3F11 |
| pSyk^Y525^ | Rabbit | WB | 1:500 | CST | 2710 |
| Syk | Rabbit | WB | 1:500 | CST | 12358 |
| pERK | Rabbit | WB | 1:500 | CST | 9101 |
| ERK | Rabbit | WB | 1:500 | CST | 9102 |
| Bcl-2 | Hamster | IP | 3 μg IP | BD | 6C8 |
| Bcl-2 | Mouse | WB | 1:500 | BD | 610539 |
| Bad | Rabbit | WB | 1:500 | Enzo | ADI-AAP-020 |
| Bid | Rat | WB | 1:500 | R&D Systems | MAB860 |
| Puma | Rabbit | WB | 1:500 | Abcam | AB9643 |
| P53 | Rat | WB | Supernatant | EuromAbNet | POE316A |
| Bax | Rabbit | WB | 1:500 | Santa Cruz | N-20 |
| Bak | Rabbit | WB | 1:500 | Santa Cruz | G-23 |

Cells were re-suspended at the required density (Eμ-Tcl1 leukemias 5x10^6^/ml, CLL 1x10^7^/ml, DLBCL cell lines 1x10^6^/ml) and treated as indicated in the figure legends. After the required time period, cells were collected, washed, and lysed using RIPA buffer (150 mM NaCl, 1% Triton X-100, 0.5% Deoxycholate, 0.1% SDS, 50 mM Tris, pH 8). Sample protein quantitation was performed using a BSA standard curve and Bradford assay quantitation (BioRad, Hemel Hempstead, UK). 50 μg protein was loaded into 10% Novex NuPage Bis Tris gels (Thermo Fisher, Loughborough, UK) and transferred to a methylcellulose membrane (GE Lifesciences, UK) by standard techniques. Antibodies utilized are detailed below.

**Immunoprecipitation**

For immunoprecipitation of Eμ-Tcl1 Tg leukemias or human DLBCL cell lines, 1x10^8^ cells per sample were lysed in CHAPS lysis buffer (20 mM Tris pH 7.4, 142.5 mM KCl, 2 mM CaCl_2_, 1% CHAPS). 500μg protein was pre-cleared by addition of 50μl 50% v/v protein G sepharose in CHAPS lysis buffer (10% total volume) for 2 hours at 4°C. Simultaneously, protein G sepharose beads were blocked with 5% normal rat serum for 2 hours at 4°C. Pre-cleared lysates and blocked beads were then pelleted and the relevant fractions retained. Pre-cleared lysates were then incubated with 50μl blocked protein G Sepharose and 5% normal rat serum for 2 hours at 4°C, after which samples were pelleted and the beads discarded. 50μl blocked protein G sepharose was incubated with a sufficient amount of antibody to give a final concentration (when applied to lysates) of 2μg/ml for 2 hours at 4°C. Lysates were then centrifuged, the bead pellet discarded and 50μl of the antibody bead mixtures added to lysates and mixed overnight at 4°C with agitation. Beads were subsequently pelleted, retained, and washed five times in lysis buffer followed by incubation with reducing 2x Laemmli buffer and boiled for 5 minutes. Lysates were then analyzed by SDS-PAGE and western blotting using VeriBlot HRP conjugated antibodies (Abcam, UK) as the secondary reagents. Primary Human CLL were treated as above, except 50 μg protein was incubated with 3 μg anti-Human Bcl-2 (6C8) in a final reaction volume of 100 μl.

**Antibodies & Inhibitors**

Goat anti-mouse IgM F(ab’)_2_ was obtained from Jackson Immunoresearch (West Grove, USA). Anti-human BCR mAbs were produced in house (anti-IgM: M15/8, anti-IgG: SB2H2). Etoposide, R406, Ibrutinib, and ABT-199 (*in vitro* use) were obtained from Selleck Chemicals (Houston, USA). ABT-199 for *in vivo* use was obtained from Active Biochem (Hong Kong, China).

**Cell viability assays**

Purified Eμ-Tcl1 Tg cells were re-suspended at 2.5x10^6^ cells/ml and incubated with the indicated concentration of inhibitor or an appropriate vehicle control for 48 hours, harvested and stained with 1 µg/ml Annexin V^FITC^ (in-house) and 3.6 µg/ml PI (Sigma, Gillingham, UK), for 15 minutes and analyzed on a FACScalibur flow cytometer (BD Biosciences, Oxford, UK). Primary human CLL were treated as above except cells were utilized at a final concentration of 5x10^6^/ml. DLBCL cell lines were utilized at a final concentration of 0.5x10^6^/ml. % viability was calculated as (% viable cells in sample / % viable cells in vehicle control) x100.

**Chemotaxis assay**

Purified Eμ-Tcl1 Tg leukemias were washed twice in low-serum medium (RPMI, 0.1% FCS) and resuspended at 1x10^7^/ml. Cells were then pre-incubated with 0.6 μM GS-9820, 1 μM R406, 1 μM Ibrutinib, or an appropriate vehicle control for 1 hour in low-serum medium. 1x10^6^ cells were loaded into the upper chamber of a 24-well 5 μm polycarbonate membrane transwell plate (Corning, Amsterdam, NED) and subjected to 200 ng/ml CXCL12 or 1 μg/ml CCL21 (Peprotech, London, UK) in low-serum medium for 3 hours at 37^o^C. Transmigrated cells were then harvested, and enumerated using AccuCheck counting beads (Thermo Fisher, Loughborough, UK) and CD5^+^ x B220^+^ flow cytometry. Samples were performed in triplicate and data is presented as fold change over medium alone. In the case of comparing the effectiveness of inhibitors between genotypes migration index was calculated. Migration index = number of CXCL12-induced transmigrated cells in sample/ number of CXCL12-induced transmigrated cells in vehicle control.

**Adhesion Assay**

Purified Eμ-Tcl1 Tg leukemias were washed and re-suspended at 5x10^6^/ml in RPMI 0.5% BSA and pre-incubated with 0.6 μM GS-9820 or an appropriate vehicle control for 1 hour at 37^o^C. Cells were then stimulated with 10 μg/ml anti-mouse IgM (Fab)’_2_ or PBS alone and plated into fibronectin-coated plates and incubated for 3 hours. Plates were washed twice and adherent cells fixed with 1% glutaraldehyde PBS. Cells were then stained for 15 minutes with 0.5% crystal violet (Sigma, Gillingham, UK), 25% methanol and washed thoroughly. 50% acetic acid was then applied and plate absorbance at 590 nm determined using an epoch microplate spectrophotometer (Biotek, Swindon, UK). Background absorbance was recorded by measuring dyed wells without cells. Non-specific binding was assessed by adherence to BSA-coated wells. Data was normalized to maximum achievable binding in PLL-coated wells by the equation adhesion in sample/ PLL-induced adhesion x 100%.

**HK Co-culture Assay**

2.5x10^5^ HK cells per well were plated in a 24-well plate and allowed to adhere overnight. Subsequently, Purified Eμ-Tcl1 Tg leukemias were re-suspended at 5x10^6^/ml and pre-incubated with 0.6 μM GS-9820 or an appropriate vehicle control for 1 hour at 37^o^C. Eμ-Tcl1 Tg leukemias were then applied to HK-cell-containing wells for 3 days. Cells were then harvested and analyzed for viability by annexin V/PI flow cytometry. During harvesting HK cells largely remained attached to tissue culture plastic. However, FSC/ SSC discrimination was also performed to prevent measurement of contaminating stromal cells.

**Cellular Adhesion Assay**

Purified Eμ-Tcl1 Tg leukemias were stained with 5 μM CFSE and re-suspended at 1x10^6^/ml in RPMI 0.5% BSA and pre-incubated with 0.6 μM GS-9820, 1 μM Ibrutinib, or an appropriate vehicle control for 1 hour at 37^o^C. Cells were then applied to collagen-coated plates (Thermo Fisher, Loughborough, UK) containing HK-cells at a leukemia:HK ratio of 20:1 for 2 hours at 37^o^C. Wells were then washed, to remove non-adherent cells, and adherent cells removed via application of Trypsin EDTA (Thermo Fisher, Loughborough, UK) for 5 minutes. Adherent cells were then harvested, washed, and analyzed by flow cytometry for the presence of CFSE^+^ cells, using AccuCheck counting beads (Thermo Fisher, Loughborough, UK) for enumeration.

**Phagocytosis Assay**

Murine or human macrophages were utilised for phagocytosis assay using Eμ-Tcl1 Tg leukemias or CLL samples, respectively. For murine studies, bone marrow-derived macrophages were produced by isolation of adherent cells derived from C57BL/6 bone marrow. Cells were cultured *in vitro* utilizing L929-conditioned media for 7 days. For human samples, monocyte-derived macrophages were differentiated from total PBMCs. PBMCs were isolated from healthy human volunteers by density gradient centrifugation and plated in RPMI supplemented with autologous serum for 2 hours. Non-adherent cells were removed and adherent cells cultured in RPMI supplemented with 100ng/ml recombinant human M-CSF (generated in house), glutamine, pyruvate, FCS, and antibiotics for 7 days. On the day prior to the assay, macrophages were harvested using trypsin/EDTA (Thermo Fisher, UK), plated in a 96-well plate at 5x10^4^/well, and left overnight to adhere. Eμ-Tcl1 Tg leukemias were purified from cryopreserved splenocyte samples by density gradient centrifugation (Axis Shield, Norway) and assessed for purity by CD5^+^ x B220^+^ flow cytometry. Purified cryopreserved primary CLL samples were obtained and thawed 1 hour prior to use. Cells were then labeled with 5 μM CFSE and pre-incubated with 0.6 μM GS-9820 or DMSO control for 1 hour. Cells were opsonized with 10 μg/ml 18B12 mouse IgG2a, 1 μg/ml rituximab (Human IgG1), or an appropriate Isotype control for 30 minutes on ice. Cells were then warmed and added to macrophages at a ratio of 5:1. Plates were pulsed in a centrifuge and incubated for 1 hour at 37°C. Cells were then stained with anti-F4/80 (Bio-Rad, UK) to distinguish macrophages and analysed by flow cytometry. Cells were gated on macrophages and the percentage of cells staining positive for CFSE and F4/80 was recorded.

**Supplementary Figures**

**Supplementary figure 1 Eμ-Tcl1 Tg mice spontaneously develop CD5^+^ B-cell leukemia. *(A)*** *Eμ-Tcl1 Tg animals were monitored for spontaneous pathology and sacrificed upon attainment of experimental endpoint criteria. Animals presented with extensive splenomegaly and modest lymphadenopathy with a median survival of 295 days.* ***(B)*** *Animals from* ***(A)*** *were assessed for spontaneous leukemia development by monthly blood sampling and CD5 x B220 flow cytometry and upon terminal disease (T) organs were harvested, enumerated, and contrasted to aged C57BL/6 animals (C57). Data represents the average of values obtained from at least 14 Eμ-Tcl1 Tg and 5 C57BL/6 animals. Error bars represent SEM. Statistical analyses were performed using an unpaired Student’s T-test. **= p<0.005, ***= p<0.0005, ****= p<0.00005, n.s = non-statistically significant. Survival data represents the average of 32 Eμ-Tcl1 Tg and 15 C57BL/6 animals. Survival analysis was performed using a log rank test.*

**Supplementary figure 2 Immunophenotype analysis of Eμ-Tcl1 Tg leukemias.** *The Immunophenotype displayed by splenocytes harvested from terminal Eμ-Tcl1 Tg animals was analyzed by flow cytometry. Cells were gated according to CD5^+^ B220^+^ staining, typically representing in excess of 80% of total splenic lymphocytes, and surface marker expression analyzed and contrasted to normal B-cell populations from aged C57BL/6 animals (C57). Data is expressed as a ratio of MFI: MFI of an appropriate isotype control. Statistical analysis was performed using an unpaired Student’s T-test. n.s = non significant, *= p<0.05, **= p<0.005.*

**Supplementary figure 3 GS-9820 exhibits pharmacokinetic properties appropriate for use in *in vivo* murine models.** GS-9820 and GS-1101 (Idelalisib) structures obtained from (8) and (54). Isoform specific Ec50 values for GS-9820 were adapted from (*8*). Briefly, for PI3Kδ and PI3Kγ Ec50s human fibroblasts were utilized and murine embryonic fibroblasts were employed for PI3Kα and PI3Kβ. Stimuli & readout: PI3Kα = PDGF-induced Akt phosphorylation, PI3Kβ = lysophosphatidic acid (LPA)-induced Akt phosphorylation, PI3Kδ = Anti-FcεRI-induced CD63 expression, PI3Kγ = *N*-formyl-methionyl-leucyl-phenylalanine (fMLP)-induced CD63 expression. Plasma concentrations of GS-9820 were assessed in female athymic nude (nu/nu) mice with OVCAR3 cell xenografts following twice daily administration of GS-9820 by oral gavage for 30 days at 10, or 30 mg/kg.

**Supplementary figure 4 GS-9820 inhibits PI3K-dependent signaling downstream of BCR and Chemokine receptors.** *Eμ-Tcl1 Tg leukemias were pre-incubated with GS-9820 at the indicated concentration, or 0.6 μM where not otherwise stated, followed by application of anti-mouse IgM (20 μg/ml) for 3 minutes* ***(A-B)*** *or CXCL12 (200 ng/ml)* ***(C)*** *for the indicated time period. Cells were then lysed and analyzed by western blot. Data are representative of at least two independent experiments utilizing different Eμ-Tcl1 Tg leukemias.* ***(D)*** *CLL: HK co-cultures were established prior to GS-9820 (0.6 μM) or DMSO control application. CLL viability was assessed 3 days later by Annexin V/PI flow cytometry. Data represents averages of 6 different patient samples and were analyzed using paired Student’s T-test. Error bars represent SEM. * = p<0.05, ** = p<0.005, *** = p<0.0005.*
**Supplementary figure 5 GS-9820-dependent Bim upregulation is evident in some but not all CLL samples. *(A-B)*** *Primary human CLL samples were cultured alongside 25 μM of the general caspase inhibitor qVD-OPH and 0.6 μM GS-9820 (GS) or a DMSO vehicle control for 48h, lysed, and analyzed for Bim expression* ***(A)*** *and co-immunoprecipitation with Bcl-2* ***(B)*** *by western blot. Upregulation of Bim expression was evident in half of all analyzed CLL and correlated with enhanced Bcl-2 co-immunoprecipitation. Changes in Bim expression did not correlate with IgHV mutation status or other prognostic markers (CD38, or Zap70). Data represent representative examples of at least 4 CLL samples.* ***(C)*** *CLL samples were cultured with 0.6 μM GS-9820 or a DMSO vehicle control for 24h prior to application of ABT-199 or a DMSO control for an additional 24h and viability assessed by Annexin V/ PI flow cytometry. Data represents an average of 4 different CLL samples. Statistical analysis was performed using two-way ANOVA. *= p<0.05, **= p<0.005, ***= p<0.0005.*

**Supplementary figure 6 GS-9820 inhibits PI3K-dependent BCR signaling in DLBCL cell lines.** *DLBCL cell lines (SUDHL-4, SUDHL-6, HBL-1, and OCI-LY10) were pre-incubated with the indicated concentration of GS-9820 followed by application of BCR stimulation (20 μg/ml) (Anti-IgG for SUDHL4, and Anti-IgM for SUDHL6, HBL-1, and OCI-LY10) for 3 minutes. Cells were harvested, lysed, and analyzed by western blot.*

**Supplementary figure 7 GS-9820-induced cellular apoptosis is co-incident with upregulation of Bim in DLBCL cell lines. *(A)*** *DLBCL cell lines were treated with the indicated concentration of GS-9820 for 48h and assessed for viability by Annexin V/PI flow cytometry. Data represent the average of three independent experiments per cell line and were normalized to a DMSO control. Error bars represent S.E.M.* ***(B-C)*** *DLBCL cell lines were pre-incubated with 25 μM qVD-OPH and subjected to the indicated concentration of GS-9820 for 48h followed by lysis and analyzed for Bim expression or Bim: Bcl-2 co-immunoprecipitation by western blot. Data is representative of two independent experiments per cell line.* ***(D)*** *DLBCL cell lines were treated with 0.6 μM GS-9820 or DMSO control for 24h prior to application of the indicated concentration of ABT-199 or a DMSO control for an additional 24h. Cells were then harvested and viability assessed by Annexin V/PI flow cytometry. Data represents an average of 3 independent experiments per cell line. Statistical analysis was performed by two way ANOVA. **= p<0.005, ***= p<0.0005,****= p<0.00005, n.s = non significant.*

**Supplementary figure 8** **Bcl-2 family expression profile of Eμ-Tcl1 Tg leukemias. *(A)*** *Eμ-Tcl1 Tg leukemias were purified from the spleens of tumor-bearing animals, lysed, and analyzed by western blot for Bcl-2 family member expression in comparison to normal C57BL/6 B-cells (C57). Data is representative of 2 independent experiments utilizing 12 different Eμ-Tcl1 Tg leukemias and 7 C57BL/6 B-cell populations in total.* ***(B Left)*** *Densitometry analysis was performed on total Bim expression levels (Bim_EL_, Bim_L_, and Bim_S_ combined), corrected for Tubulin expression, and normalized to normal C57BL/6 B-cell populations.* ***(B Right)*** *Relative Bim expression levels were correlated with the maximum levels of GS-9820-induced cell death achieved (10 μM).* ***(C)*** *Purified Eμ-Tcl1 Tg leukemias and normal B-cells from WT (C57BL/6) and Puma^-/-^ animals were cultured ex vivo for 6h in the presence of 20 μg/ml Etoposide (Etopo) or a DMSO control, lysed, and analyzed by western blot for Puma and p53 expression levels.*

**Supplementary figure 9** ***Bim^-/-^*** **Eμ-Tcl1 Tg leukemias do not phenotypically differ from their WT counterparts. (A)** *Eμ-Tcl1 Tg leukemias from Bim^+/+^, Bim^+/-^, or Bim^-/-^ backgrounds were purified, lysed, examined for Bcl-2 family member expression profiles by western blot, and compared to normal C57BL/6 B-lymphocytes from a Bim^+/+^ or Bim^-/-^ background.* ***(B)*** *Eμ-Tcl1 Tg leukemias from a Bim^+/+^ or Bim^-/-^ background were purified and assessed for surface CD38 and CD23 staining by flow cytometry. Cells were gated according to CD5+ B220+ staining and data is expressed as a ratio of MFI: MFI of an appropriate isotype control. Data represents an average of at least 10 different Bim^+/+^ and 6 Bim^-/-^ Eμ-Tcl1 Tg leukemias and 3 C57BL/6 B-cell populations. Statistical analysis was performed using an un-paired Student’s T-test. n.s= non significant.*

**Supplementary figure 10 Overexpression of Bcl-2 abrogates GS-9820- and Ibrutinib-induced cell death in Eμ-Tcl1 Tg leukemias.** *Eμ-Tcl1 Tg animals were crossed onto a Vav-Bcl-2 Tg background and monitored for disease presentation (manuscript in preparation). Leukemia bearing animals were sacrificed and leukemia populations purified from the spleens of afflicted animals. Eμ-Tcl1 Tg leukemias from Bim^+/+^, Bim^-/-^ or Vav-Bcl-2 Tg backgrounds were cultured in the presence of the indicated concentration of GS-9820 or Ibrutinib for 48h and viability assessed by Annexin V/PI flow cytometry. Statistical analysis was performed using two-way ANOVA analysis. *= p<0.05, **= p<0.005.*

**Supplementary figure 11 in vivo administration of GS-9820 does not induce toxicity or affect anti-CD20-mediated leukemia cell depletion (A)** *SCID animals were inoculated with Eμ-Tcl1 Tg leukemias and monitored for disease presentation. Upon detection of leukemic cells in the blood, animals were randomized to receive either 10 mg/kg GS-9820 per os BID or an appropriate vehicle control and body weight monitored weekly. Data represents the average of 4 different Eμ-Tcl1 Tg leukemias of both Bim^+/+^ and Bim^-/-^ backgrounds (Light symbols represent data obtained from Bim^-/-^ Eμ-Tcl1 Tg leukemia recipient animals). Data is expressed as % Change in body weight from pre-treatment.* ***(B)*** *Wild-type (WT) or FcγR γ chain^-/-^ (γ^-/-^) animals were inoculated with Eμ-Tcl1 Tg leukemias and upon presentation were treated with (250 μg) anti-mouse CD20 mAb (Clone: 18B12 IgG2a) (n=3 per group) and blood sampled 48h later. Leukemic burden was normalized to pre-treatment levels.* ***(C)*** *SCID mice were inoculated with Bim^+/+^Eμ-Tcl1 Tg leukemias and randomized into treatment groups (n=5 per group) receiving GS-9820, ABT-199 (50-100 mg/kg), combinations of both, or a vehicle control and body weight monitored To assess initial sensitivity, animals were dosed with 50 mg/kg ABT199 for 7 days and escalated to 100 mg/kg for a further 14 days. Statistical analyses were performed using an upaired Student’s T-test or two-way ANOVA ***= p<0.0005.*

 **Supplementary figure 12 GS-9820 administration does not affect *in vivo* MDSC frequency.** *SCID mice* *were inoculated with 1x10^7^* *Eμ-Tcl1 Tg leukemias and randomized into vehicle or GS-9820 treatment groups once CD5^+^ tumor B-cells were detected. Animals were treated with 10 mg/kg GS-9820 per os BID or a vehicle control until terminal disease criteria were met. Animals were sacrificed and the MDSC content of splenocytes was assessed by flow cytometry using CD11b, Ly6C, and Ly6G markers representative data shown in* ***(A)****. The extent of MDSC populations was then assessed between GS-9820 and vehicle recipient animals* ***(B)****. Data was obtained from both Bim^+/+^ (Black squares) and Bim^-/-^ (Grey squares) Eμ-Tcl1 Tg leukemia-recipient animals. Data were analyzed by an un-paired Student’s T-test. *** = p<0.0005, n.s= non significant.*
